# Clinical efficacy of the mulligan maneuver for cervicogenic headache: a randomized controlled trial

**DOI:** 10.1038/s41598-023-48864-1

**Published:** 2023-12-12

**Authors:** Xin Jin, Hong-Gen Du, Ning Kong, Jian-Liang Shen, Wen-Jun Chen

**Affiliations:** 1 Department of Tuina, The First Affiliated Hospital of Zhejiang Chinese Medical University, Hangzhou, 310000 China; 2 Department of Radiology, The First Affiliated Hospital of Zhejiang Chinese Medical University, Hangzhou, 310000 China; 3 Hangzhou Medical College, Hangzhou, 310000 China

**Keywords:** Neuroscience, Neurology, Neurological disorders

## Abstract

Cervicogenic headache is an often observed secondary headache in clinical settings, with patients who endure prolonged and persistent pain being particularly susceptible to mood changes. Currently, the Mulligan is one of the effective methods for CEH. However, there is a lack of evaluation about the strength and frequency of headaches, as well as the assessment of pain-induced emotions, in individuals with CEH using this particular procedure. Herein, we aimed to evaluate the effectiveness of the Mulligan maneuver from a multidimensional perspective of pain intensity and mood. A total of forty patients diagnosed with CEH who satisfied the specified inclusion criteria were recruited and allocated randomly into two groups: the control group and the treatment group, with each group consisting of twenty cases. The control group received health education, while the treatment group received the Mulligan maneuver once daily over a course of 10 treatment sessions.The clinical outcome of patients with CEH in two groups was assessed using the Visual Analog Scale (VAS), Hamilton Anxiety Scale (HAMA), and Hamilton Depression Scale (HAMD). Resting-state functional magnetic resonance imaging was employed to examine variations in brain function activities between the two CEH groups. Brain regions showing differences were identified as regions of interest and subsequently correlated with clinical behavioral measures using Pearson’s correlation analysis. The differences in VAS, HAMA and HAMD between the two groups of CEH patients were also statistically significant. The brain regions that showed differences in the ReHo scores between the two groups of CEH patients included the left cerebellum, the frontal gyrus, and the middle temporal gyrus. There was a positive correlation between the left frontal gyrus and VAS, HAMA and HAMD. The left middle temporal gyrus had a negative correlation with VAS, HAMA, and HAMD and the left cerebellum had a positive correlation with VAS correlation. The Mulligan maneuver may improve pain levels and have a moderating effect on pain-related negative emotions by regulating the function of relevant brain regions in CEH patients.

## Introduction

Cervicogenic headache (CEH) is one of the most common secondary headaches in clinical practice and is caused by bony, disc, or soft tissue disorders of the cervical spine^1^. CEH is a form of entrapment pain and explained its pathogenesis with the congregation theory. According to this theory, lesions in the structures innervated by the high cervical nerves (the greater occipital, lesser occipital, and greater auricular nerves belong to the second and third cervical nerves) cause afferent injurious sensory messages from the high cervical nerves that are connected^2^. The prevalence of cervicogenic headache was estimated at 1%, 2.5%^3^ or 4 0.1%^4^ of the total population and up to 17.5% in patients with severe headaches^3^. The Prevalence is up to 53% in patients with post-whiplash headache^5^. The clinical diagnostic criteria include unilateral headache with evidence of cervical involvement by provocation of pain by neck motion or pressure on the neck; concomitant pain in the neck, shoulder and arm; and reduced neck range of motion, with or without other features^6^. Approximately 54% of CEH patients can develop chronic intractable pain. This can lead to anxiety or depression, seriously affecting the daily activities of patients^7^.

Recent studies have shown that manipulation can positively affect headache intensity, frequency, and cervical spine dysfunction in CEH patients^8^. Based on a systematic review and meta-analysis, we found that manipulation can significantly improve the symptoms of head and neck pain in CEH patients and restore normal physiological function of the cervical spine without obvious side effects^9^. However, manipulation has not been reported to improve pain cognition or pain emotion in CEH patients. Recent studies have shown that the manipulation has a modulatory effect on the functional activity of certain regions of the brain. The manipulation can activate the function of the bilateral orbito gyrus, the middle frontal gyrus, the bilateral cuneate lobes, the bilateral inferior frontal gyrus, and the bilateral anterior cingulate gyrus. It increases connectivity between the sensory and executive cortex and inhibits its connection to the cognitive, visual, and memory cortex^10,11^.

The Mulligan manoeuvre is one of the best manipulations. Unlike traditional mobilisation, which relies solely on the therapist, Mulligan posited that by exerting pressure on the spinous processes in a weight-bearing stance, the facet joints would undergo a synchronized sliding motion in a parallel manner. At the same time, It requires the patient to move actively in order to achieve the perfect therapeutic effect. After more than half a century of clinical practice and technical improvement, it has formed a safe and effective mature treatment for cervical and upper thoracic spine disorders. The Mulligan maneuver is highly effective in the manipulative treatment of CEH.

A systematic review have demonstrated the efficacy of the Mulligan maneuvre for CEH on clinical pain scales and cervical range of motion after treatment^12^. However, they do not take into account the fact that chronic pain results in feelings of anxiety or depression in patients. The Mulligan maneuver has been innovatively proposed not only for CEH pain symptoms, but also for emotional problems due to chronic CEH pain in our study. In order to demonstrate the therapeutic effect of the Mulligan manoeuvre for CEH, clinical rating scales were used to demonstrate its therapeutic effect on pain and its induced mood, and fMRI technology was innovatively introduced to demonstrate its therapeutic effect on pain and emotion-related brain areas.

## Methods/design

This study used a fully randomized, controlled, double-blinded, double-si-mulation research method. The study was in accordance with the Consolidated Standards of Reporting Trials (CONSORT) checklist guidelines. The study was registered in the China Clinical Trial Registration Center (Registration Number: ChiCTR2100054072 23/08/2021) and reviewed and approved by the Ethics Committee of the first affiliated hospital of Zhejiang Chinese Medical University with approval number (Ethics Number: 2021-K-491–01 19/07/2021). All subjects signed an informed consent before the study. All methods were carried out in accordance with the tenets of the Declaration of Helsinki as well as the relevant guidelines and regulations.

Eligibility criteria: All participants were diagnosed with CEH according to the diagnostic criteria for CEH in the third edition of the International Classification of Headache Disorders (ICHD-3) in 2018. They were examined at the outpatient clinic of the first affiliated hospital of Zhejiang Chinese Medical University from January 2022 to December 2022.

Inclusion criteria were as follows: CEH pain intensity 3–8 on 10-point pain scale; Cervical spine dysfunction; Cervical motion reduction; Headache after neck pain; Patients with neck tightness and limited range of motion.

Exclusion criteria: History of cervical spine surgery or history of severe cervical spine trauma; Combination of severe osteoporosis, bone tumor, and bone tuberculosis; Combination of cardiovascular, cerebrovascular, digestive, hematologic and other serious medical diseases; Combined with mental diseases, such as claustrophobia and mental retardation; Pregnant or lactating women; People with language comprehension and expression disorders; Contraindications to magnetic resonance, such as metal foreign bodies in the body; and pacemaker implantation.

Dropout Criteria: Cases that have been enrolled but have not completed the clinical program should be considered to have dropped out in the following cases: The patient withdraws from the study on his/her own; Subjects with poor compliance cannot come for treatment as required, and the patient does not cooperate; The treatment is effective during treatment but cannot complete the entire observation program.; The treatment is effective during treatment, but the patient cannot complete the entire observation program; The subject does not withdraw from the study but no longer accepts the treatment and loses the visit; In the course of the study, other diseases occur, and the treatment is stopped for more than one week in the middle of the study.

### Procedures

First, all participants signed an informed consent form with the right to withdraw at any time. After recruitment, a qualified therapist will explain the study procedures and collect demographic data at the initial assessment following recruitment. Throughout the study, confidentiality will be maintained.

**Figure 1:**
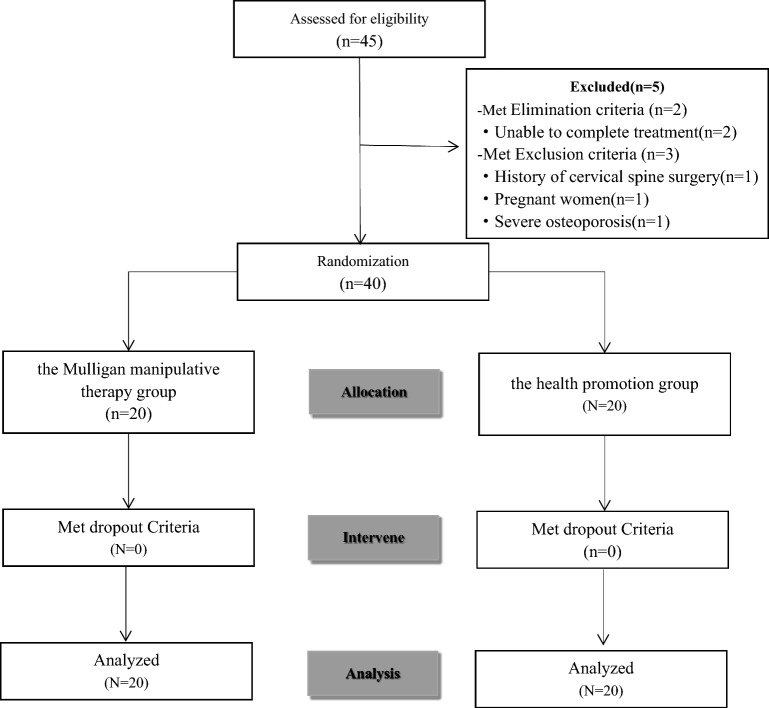
Consolidated Standards of Reporting Trials (CONSORT) diagram showing the enrollment patients in this study.

Participants were assigned to the Mulligan manipulative therapy group or the health education group in a 1:1 ratio. Randomisation was computer generated centrally by the Clinical Evaluation Centre of the first affiliated hospital of Zhejiang Chinese Medical University, China. The randomization method achieved allocation concealment using randomization envelopes that were opaque and sealed randomization envelopes. The allocation scheme of two groups was placed in envelopes with the serial number of the group on the outside and the group printed on the inside of the envelope. The envelopes with numerical identifiers were sequentially unsealed in accordance with the order of patients who were still not being attended to and the administration of treatment was carried out according to the specified interventions outlined within the envelopes and treatment was administered according to the interventions requested within the envelopes. The treatment regimen received by each subject was determined by the generated randomized sequence. The Clinical Evaluation Centre of the first affiliated hospital of Zhejiang Chinese Medical University was responsible for the efficacy evaluation to achieve blinding of patients and statistical evaluators. A sample expansion of 20% was proposed, given the possibility of attrition in the study (Fig. 1).

### Interventions

#### Mulligan maneuver therapy group

**Figure 2:**
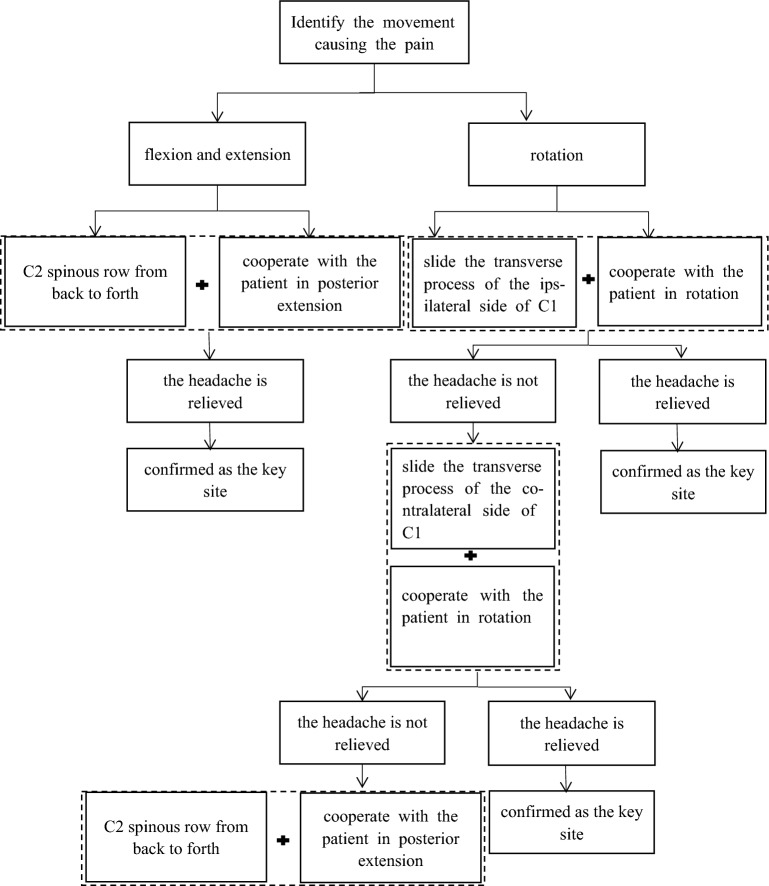
Confirm the key site flowchart. *Note* Areas of decreased ReHo in CEH patients in the Mulligan manoeuvre treatment group compared to the health promotion intervention group include middle temporal gyrus (blue areas); areas of increased ReHo include cerebellum, middle frontal gyrus (red areas).

The team used the FingerTps(made in USA) device to measure the pressure exerted by the operator. FingerTps is worn on the operator’s thumb and the information collected about thumb pressure is transmitted in real time to a computer via a wireless transmitter in the finger sleeve, which allows the operator to adjust the force using a display screen^13^. On the day of enrolment, CEH patients underwent the Mulligan Maneuver individually and in person at the tuina department of the Zhejiang Provincial Hospital of Traditional Chinese Medicine. The Maneuver used were carried out by a therapist possessing over six years of expertise who underwent training in both the subject matter of investigation and quality control before the beginning of the trial. This study used the “Assessment-treatment-re-assessment” treatment protocol^8^. The intensity of pain and the perception of pain in patients with CEH were assessed by an independent assessor on the day of enrolment.The therapist positioned the key therapeutic sites of CEH patients according to the process (Fig. 2).The specific procedures are as set out below for flexion–extension or rotational headaches. Flexion–extension headache: In the seated position, the operator applies a constant posterior-anterior thrust at the assessed site of application (posterior border of the C2 spinous process), This thrust is accompanied by a compressive force of approximately 50 N, directed towards the patient’s eyes. The operator instructs the patient to perform a cervical lordosis maneuver while the remaining fingers of the therapist immobilize the patient’s cheeks. This procedure is repeated three times. Rotational headache: In the seated position, the operator applied a steady posterior-anterior thrust at the assessed site of application (left or right side of the C1 transverse process), maintaining a compressive force of approximately 50 N and directed towards the patient’s eyes. Simultaneously, the patient was instructed to turn his head to the same side, and the procedure was repeated three times. If the patient feels any discomfort such as aggravated headache, nausea or vomiting during the manipulation, he should stop the manipulation immediately. The manipulation schedule, once per day, ten times for one session. At the end of one session of treatment, the CEH patients were re-assessed by an independent rater for pain intensity and pain sensation, and MRI scans were performed without delay.

#### Health promotion intervention group

The research group used WeChat group building to carry out the intervention with the CEH patients. On the day of enrolment, the CEH patients’ pain intensity and pain emotion were evaluated by an independent evaluator. After the evaluation, the staff will send the health education content to the we chat group on a regular basis, which includes the following information such as the definition of CEH, the primary causes of diseases, daily preventive measures, lifestyle habits, and dietary recommendations. It emphasizes the importance of avoiding excessive strain, exposure to wind and cold, and prolonged usage of electronic devices such as computers and mobile phones. However, it does not encompass the treatment of cervical vertebrae or exercise methods for rehabilitation within the scope of health education. The process is considered complete when the patient responds to the message in the group. Staff can call the patient to complete the reading to respond if the patient does not respond to the message. The message is sent out once a day, and ten times for each course of treatment. After one treatment session, the CEH patients were reassessed by an independent assessor for pain intensity and pain sensation, and MRI scans were performed immediately.

### Image acquisition

A GE Discovery MR750 3.0T MRI scanner was used for imaging. The subjects underwent structural and BOLD functional imaging, and the scanning range was from the frontal-parietal to the base of skull, covering the entire brain. The scanning parameter settings: The structural image was acquired using the weighted fast scrambled phase gradient echo sequence technique. This allowed for acquiring the axial weighted images of T1W1 and T2W1. The scanning parameters were as follows: a repetition time of 1900 ms, a field of view set to 256 × 256 cm, an echo time of 2.26 ms, a matrix size of 256 × 256, and a scanning time of 7 min. Additionally, BOLD functional imaging was performed using the gradient echo sequence technique with a single excitation planar echo to acquire the information function. Scanning parameters: The repetition time is 2000 ms, the time point is 240, the echo time is 30 ms, the number of layers is 30, the layer thickness is 5 mm, the matrix is set to 64 × 64, the field of view is set to 256 × 256 cm, the flip angle is 90°, and the scanning time is 8 min.

### Data preprocessing

Preprocessing was performed using the Statistical Paretric Mapping12 (SPM12) software package based on the MATLAB R2014a platform, which mainly includes removing time points, temporal layer correction and head movement correction, spatial normalization, de-linearization drift, and noise filtration. After preprocessing, the data were used to calculate the local consistency of each voxel with its neighboring 27 voxels in the same time series by applying Kendall’s concordance coefficient. After preprocessing, Kendall’s concordance coefficient was applied to calculate the local coherence between each voxel and its neighboring 27 voxels in the same time series. Subsequently, the whole-brain ReHo images of subjects’ whole brains were generated. Finally, the whole-brain ReHo images were spatially smoothed using a smoothing kernel with a full-width and half-height of 6 mm to filter out the noise.

### Outcome

The primary outcomes were assessed by an assessor blinded to group allocation and intervention in the department at each time point, such as at baseline and after ten sessions of treatment. The secondary outcomes were assessed by a technician trained in magnetic resonance operation, but blinded to group allocation and intervention after patients had completed ten sessions of treatment.

#### Primary outcomes

CEH pain: A 10-point visual analogue scale (VAS) was used to measure the intensity of CEH pain^14^. Participants were asked to rate the intensity of pain on a 10 cm scale, where0 being ‘no pain’ and 10 being ‘maximum pain that is unbearable’. This is a valid and reliable (ICC = 0.60–0.77) instrument for measuring CEH pain intensity and was administered at baseline and after ten sessions of treatment.

CEH emotion: Hamilton Anxiety Scale (HAMA) scale was used to assess anxiety symptoms^15^. A scale of psychological and physical anxiety was completed by participants, ranging from 0 to 4, indicating no symptoms to extremely severe symptoms. Hamilton Depression (HAMD) scale was used to assess the depression symptoms^15^. Symptoms were scored on a five-point scale, where 0 to 4 ranged from no symptoms to extremely severe symptoms and included 7 subscales: anxiety/somatisation, weight, cognitive impairment, diurnal variation, blockage, sleep disturbance, and hopelessness.The test–retest reliability was between 0.65 and 0.91 ,and the construct validity was good.

#### Secondary outcome

The local brain functional activity: Regional Homogeneity (ReHo) is a measure of the local coherence of spontaneous brain activity and is sensitive to the following abnormal local functional connectivity of brain regions^16^. ReHo combined with clinical variables has been used to study pain and psychiatric disorders.

### Sample size calculation

This study was a randomized controlled trial where in two groups were established based on the pre-experiment outcomes. The sample size for this study was calculated based on the primary outcome of visual analogue scale. According to the literature^17^, the sample size estimation formula for comparing the two sample rates is as follows:

$$ {\text{Sample}}\,{\text{size}} = \frac{{2{\text{SD}}^{2} \left( {{\text{Z}}_{\alpha /2} + {\text{Z}}_{\beta } } \right)^{2} }}{{{\text{d}}^{2} }} $$

SD—Standard deviation = pre-experiment outcomes; Z_a/2_ = Z_0.05/2_ = Z_0.025_ = 1.96 (From Z table) at type I error of 5%; Z_β_ = Z_0.20_ = 0.842 (From Z table) at 80% power, d = effect size = difference between mean values.

The allocation of participants into the test and control groups followed a 1:1 ratio. Each group initially consisted of 16 cases, and the sample size was increased by 20% to account for potential factors including loss of visits and attrition. Consequently, a minimum of 20 cases per group was required for the study subjects.

### Statistical analysis

Data were statistically analyzed using the SPSS26 software package (SPSS Inc. USA). Measurement data was described using $\overline{x }$ ± s and count data were described using constitutive ratios and frequencies. Independent samples t-test and paired rank sum test (Z-test) were used to compare the differences in indicators between the two groups of CEH patients. The RESTplusv1.24 software package was used for statistical analysis of the data. The smReHo plots of CEH patients in the two groups were subjected to paired samples t-test, corrected for multiple comparisons by the GRF method (*P* < 0.01 for voxel level, *P* < 0.05 for clusters). The ReHo values of different brain regions were extracted and analyzed by Pearson correlation analysis with clinical behavior indices (VAS, HAMA, HAMD), and the differences were considered statistically significant at *P* < 0.05.

## Results

### Group characteristics

The clinical experiment involved a cohort of 40 patients, with 20 assigned to the Mulligan maneuver therapy group and the remaining 20 assigned to the health promotion intervention group. There were no statistically significant differences in the general age, sex, education level, disease duration, VAS score, HAMA score, and HAMD score of the patients in the two groups before the trial (*P* > 0.05) (Table 1).

**Table 1:** The demographics and characteristics of study patients before treatment were compared between the two groups.

Characteristics	Mulligan manoeuvre treatment group (n = 20)	the health promotion intervention group (n = 20)	*P* value
Age(years), Mean(SD) [CI]	30.05 (6.75) [26.84;33.36]	27.19 (4.76) [24.6;29.78]	0.15
Gender, n(%)			
Male	15 (75)	7 (35)	
Female	5 (25)	13 (65)	
Education (years), Mean (SD) [CI]	7.84 ± 1.06 [7.27;8.45]	8.91 ± 1.81 [7.98;9.84]	0.06
Course of disease (years), Mean (SD) [CI]	4.53 ± 2.65 [3.06;6.0]	4.99 ± 2.62 [3.46;6.33]	0.63
VAS scores, Mean (SD) [CI]	4.96 ± 0.89 [3.91;5.84]	5.31 ± 0.6 [4.56;5.76]	0.15
HAMA scores, Mean (SD) [CI]	21.64 ± 1.69 [19.95;22.59]	22.44 ± 1.56 [21.95;23.52]	0.13
HAMD scores, Mean (SD) [CI]	24.23 ± 1.58 [22.15;25.83]	23.64 ± 1.67 [21.78;24.97]	0.26

A* p* value < 0.05 indicates statistical significance.

### CEH pain results

According to the test of normality, the scores of the VAS scale of the two groups were in accordance with a normal distribution. After ten sessions of treatment, CEH VAS score 1.16(95% CI 0.49 to 1.82) improve (*p* = 0.00) in the Mulligan maneuver therapy group more than in the health promotion intervention group (Table 2).

**Table 2:** Comparison of VAS, HAMA and HAMD scores of CEH patients in the two groups before and after treatment.

Clinical variables	Mulligan manoeuvre treatment group (n = 20)	the health promotion intervention group (n = 20)	*p* value
VAS scores, Mean(SD)			
Before [CI]	4.96 ± 0.89 [3.91; 5.84]	5.31 ± 0.60 [4.56; 5.76]	0.15
After [CI]	3.42 ± 0.89 [3.21; 3.68]	4.58 ± 1.18 [4.21; 4.81]	0.00
HAMA scores			
Before [CI]	21.64 ± 1.69 [19.95; 22.59]	22.44 ± 1.56 [21.95; 23.52]	0.13
After [CI]	20.17 ± 1.37 [18.87; 21.75]	21.25 ± 1.71 [20.81; 21.53]	0.03
HAMD scores			
Before [CI]	24.23 ± 1.58 [22.15; 25.83]	23.64 ± 1.67 [21.78; 24.97]	0.26
After [CI]	20.79 ± 2.24 [19.82; 21.08]	22.41 ± 1.65 [21.93; 23.08]	0.01

A* p* value < 0.05 indicates statistical significance.

### CEH emotion results

After testing for normality, the Hamilton Anxiety Scale (HAMA) and the Hamilton Depression (HAMD) scale scores of the two groups were found to follow a normal distribution. After ten sessions of treatment, HAMA score 1.08 (95% CI 0.08816 to 2.072) improve (*p* = 0.03) and HAMD score 1.62 (95% CI 0.3606 to 2.879) improve (*p* = 0.01) in the Mulligan maneuver therapy group more than in the health promotion intervention group (Table 2).

### The local brain functional activity

**Figure 3:**
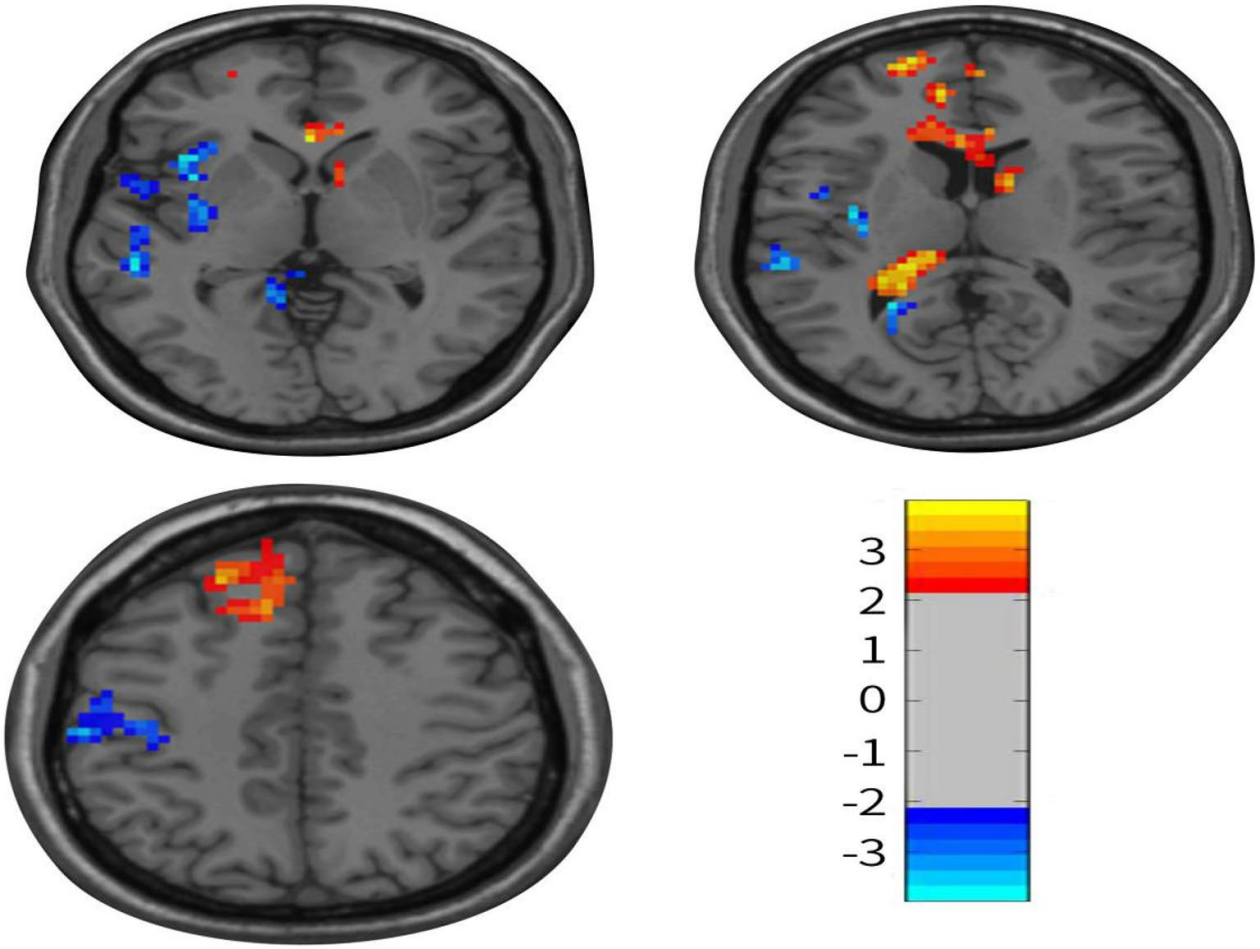
Axis of change in ReHo values in CEH patients after treatment in the Mulligan manoeuvre treatment group compared to the health promotion intervention group.

**Figure 4:**
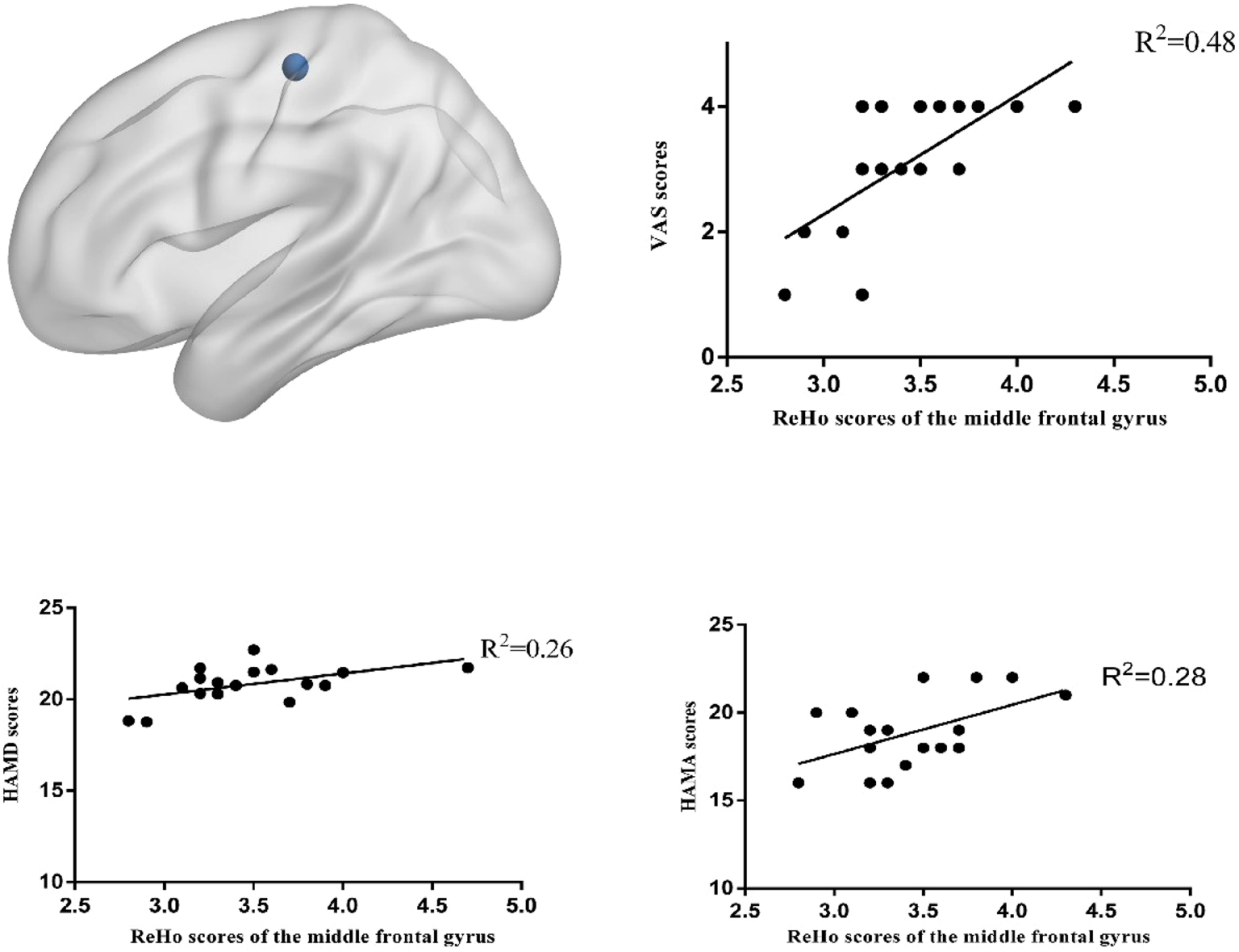
Pearson’s Correlation Analysis of the Frontal Middle Gyrus with VAS scores, HAMD scores, HAMA scores in Patients with CEH after Mulligan manoeuvre treatment.

**Figure 5:**
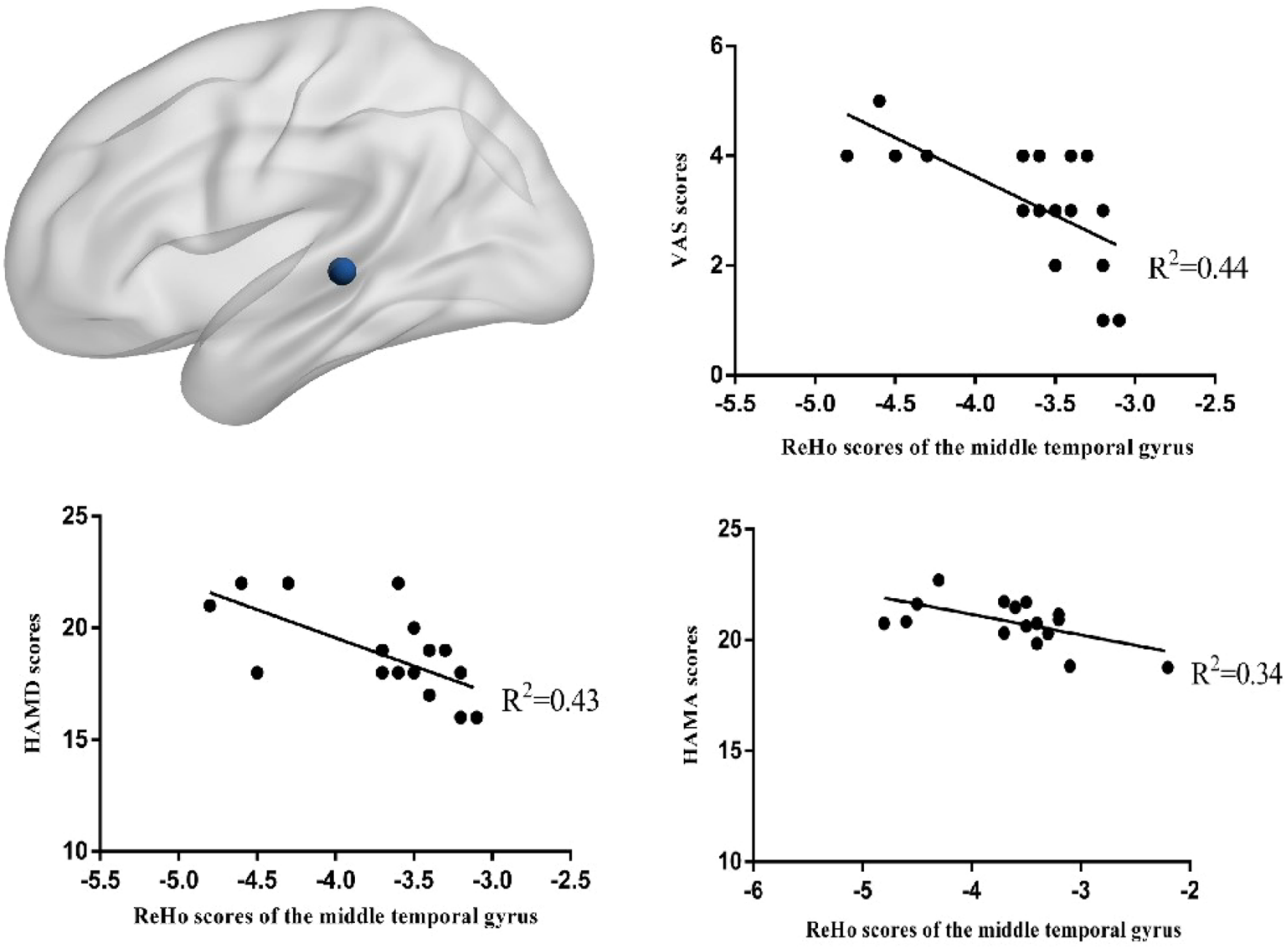
Pearson’s Correlation Analysis of the left temporal middle gyrus with VAS scores, HAMD scores, HAMA scores in Patients with CEH after Mulligan manoeuvre treatment.

**Figure 6:**
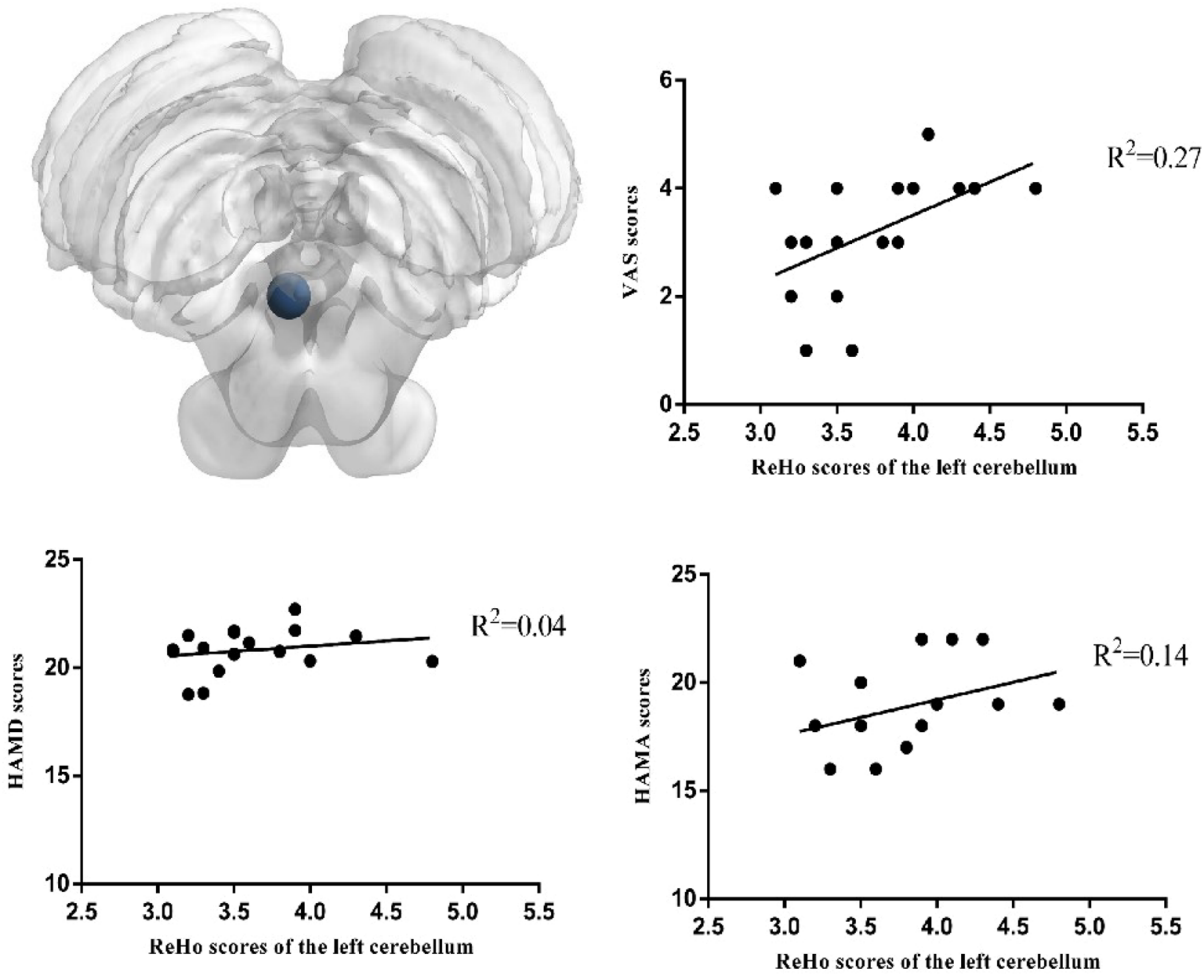
Pearson’s Correlation Analysis of the the left cerebellum with VAS scores, HAMD scores, HAMA scores in Patients with CEH after Mulligan manoeuvre treatment.

Moreover, there were differences in the changes in ReHo scores of the left cerebellum, left middle frontal gyrus, and left middle temporal gyrus between the two groups (*P* < 0.01) (Fig. 3) (Table 3). Furthermore, we observed a positive correlation between the ReHo values of left frontal gyrus and the VAS (R^2^ = 0.48, *P* = 0.00), HAMA (R^2^ = 0.28, *P* = 0.03), and HAMD (R^2^ = 0.26, *P* = 0.03) scores in the treatment group (Fig. 4). Conversely, in the treatment group, we observed a negative correlation between the ReHo values of left temporal middle gyrus and the VAS (R^2^ = 0.44, *P* = 0.00), HAMA (R^2^ = 0.43, *P* = 0.00), and HAMD (R^2^ = 0.25, *P* = 0.03) score(Fig. 5). The ReHo value of the cerebellum in the treatment group was positively correlated with the VAS score (R^2^ = 0.27, *P* = 0.03). However, no significant correlations were found between HAMA (R^2^ = 0.14,* P* = 0.13) and the HAMD scores (R^2^ = 0.04,* P* = 0.4) (Fig. 6).

**Table 3:** Regions with abnormal ReHo scores in CEH patients in comparison between two groups after treatment.

Brain area	Voxel size	Peak t-value	MNI coordinates		
			X value	Y value	Z value
ReHo scores					
Cerebellum	714	3.93	− 3	− 39	− 15
Middle temporal gyrus	1407	− 3.99	− 54	− 30	0
Middle frontal gyrus	943	3.92	− 27	21	57

## Discussion

Chronic persistent pain can lead to cognitive abnormalities in pain, subsequently causing anxiety or depression. Recent research shows that CEH patients suffer from the physical discomfort of pain, cognitive dysfunction, and psychological dysphoria^18^. A previous study has shown that CEH patients suffer from physical pain, cognitive dysfunction, and psychological distress. Our research supports this view. In addition to the higher VAS score, the HAMA and HAMD score of the CEH patients was also higher than that of the normal patients. This is an indication of depression or anxiety. Previous studies have demonstrated the effectiveness of manipulative therapy in terms of headache index, trigger point pressure pain, neck function assessment index, and range of motion in CEH^19^, and the evaluation of pain-induced emotions is less involved. The results showed: the VAS score, the HAMA and HAMD score scores decreased after the Mulligan maneuver treatment and were better than in the control group. This suggests that the Mulligan manoeuvre has a therapeutic effect on the intensity of pain, and also has some alleviating effect on pain-induced anxiety or depression.

Moreover, an additional study has shown that CEH patients have synergistic increases or decreases in neuronal activity in localized brain tissue, suggesting that CEH involves pathological changes in multiple brain regions associated with regulating affective cognition^20^. Our study is an innovative use of fMRI technology to explore the functional areas of the brain where the Mulligan maneuver has therapeutic effects, thus providing a scientific basis for the Mulligan maneuver treatment of CEH. This method has a certain uniqueness.

Herein, we found statistically significant changes in the function of frontal, middle gyrus, and middle temporal gyrus brain regions in CEH patients using the Mulligan maneuver. Furthermore, we identified correlations between these changes and clinical behavioral indicators. The middle frontal gyrus is a part of the frontal lobe that is thought to play a crucial role in regulating affective cognition, including memory and executive function. It is important to reduce pain sensitivity through affective cognitive modulation^21^. In conjunction with the results of this project, we believe that CEH patients exposed to repeated painful stimuli who are subjected to recurrent noxious stimuli experience specific aberrations in the perception and modulation of pain-related data. Several below-threshold pain tends to be amplified and processed. This is related to the fact that long-term chronic pain tends to cause damage to the frontal gyrus and emotional cognitive decline in patients with CEH. The Mulligan maneuver can somewhat improve the functional changes in the frontal gyrus and other brain regions and alleviate pain sensitivity due to cognitive decline.

The middle temporal gyrus is also a target of the Mulligan maneuver. Several studies have reported that the middle temporal gyrus is associated with emotional and sensory processing, including depression, anxiety, and pain ratings. Furthermore, it has been observed that enduring unpleasant emotional experiences resulting from pain can potentially create alterations in brain functionality. Herein, ReHo scores in the middle temporal gyrus were reduced and negatively correlated with VAS, HAMA, and HAMD, possibly due to the Mulligan maneuver’s protective inhibitory effect on the middle temporal gyrus. MEDINA^22^ study has found that cerebral blood flow in the middle temporal gyrus was reduced after treating CEH patients and Mohamadi^23^ study also shows that Manipulation affects central sensitization which can change brain metabolic map. This is consistent with our findings. So we hypothesise that by using the Mulligan technique, sensory inputs can be sent to the CNS. Perhaps these additional sensory inputs play a role in these inhibitory effects and we believe that the Mulligan maneuver can regulate the pain level and play a significant role in pain-induced emotions.

Additionally, we found that the manipulation had a benign moderating effect on changes in cerebellar function and a positive correlation between this change and VAS scores. Previous studies have focused on the cerebral cortex at the expense of cerebellar function. In a rat model of neuropathic pain, cerebellar activity was positively correlated with the development of neuropathic pain and prognosis^24^. The cerebellum plays an important role in processing pain sensations, as signal inputs from the A-fibre and C-fibre nociceptors reach the Purkinje cells of cerebellum^25^. The cerebellum is closely connected to the brain’s limbic system, amygdala, hippocampus, anterior cingulate gyrus, and frontal and temporal lobes. According to our findings, we believe that the Mulligan maneuver adjusts the neural circuits between the cerebellum and the brain by modulating the functional areas of cerebellum, thereby activating the corresponding functional areas of brain and ultimately realizing the intervention of pain emotion.

## Limitations and suggestions

Nevertheless, it is important to acknowledge the constraints of this investigation, namely the relatively small sample size and the utilization of imaging data solely for the purpose of examining alterations in regional brain functionality among CEH patients. Consequently, further investigations need to be conducted to increase the sample size and explore the underlying mechanisms pertaining to the network connectivity of local cerebral activity.

## Conclusions

Herein, we found that the Mulligan maneuver can improve pain levels and regulate pain-induced negative emotions by modulating the function of relevant brain regions in CEH patients.

## Data Availability

The data sets that were used and/or analysed in the current study are available from the corresponding author upon reasonable request.

## References

[1] Olesen D, Bes A, Kunkel R (2018). Headache classification committee of the international headache society (IHS) the international classification of headache disorders, 3rd edition. Cephalalgia.

[2] Bogduk N (2001). Cervicogenic headache: Anatomic basis and pathophysiologic mechanisms. Curr. Pain Headache Rep..

[3] Evers S (2008). Comparison of cervicogenic headache with migraine. Cephalalgia.

[4] Sjaastad O (2008). Cervicogenic headache: Comparison with migraine without aura; Vågå study. Cephalalgia.

[5] Lord SM, Barnsley L, Wallis BJ (1994). Third occipital nerve headache: A prevalence study. J. Neurol. Neurosurg. Psychiatry.

[6] Bogduk N, Govind J (2009). Cervicogenic headache: An assessment of the evidence on clinical diagnosis, invasive tests, and treatment. Lancet Neurol..

[7] Xiao H, Peng B, Ma K (2019). The Chinese association for the study of pain (CASP): Expert consensus on the cervicogenic headache. Pain Res. Manag..

[8] Satpute K, Bedekar N, Hall T (2021). Effectiveness of Mulligan manual therapy over exercise on headache frequency, intensity and disability for patients with migraine, tension-type headache and cervicogenic headache - A protocol of a pragmatic randomized controlled trial. BMC Musculoskelet. Disord..

[9] Jin X, Du HG, Qiao Z (2021). The efficiency and safety of manual therapy for cervicogenic cephalic syndrome (CCS): A systematic review and meta-analysis. Medicine (Baltimore).

[10] Wen Y, Chen XM, Jin X (2022). A spinal manipulative therapy altered brain activity in patients with lumbar disc herniation: A resting-state functional magnetic resonance imaging study. Front. Neurosci..

[11] Chen XM, Wen Y, Chen S (2023). Traditional Chinese manual therapy (Tuina) reshape the function of default mode network in patients with lumbar disc herniation. Front. Neurosci..

[12] Núñez-Cabaleiro P, Leirós-Rodríguez R (2022). Effectiveness of manual therapy in the treatment of cervicogenic headache: A systematic review. Headache.

[13] David JP, Helbig T, Witte H (2023). SenGlove-a modular wearable device to measure kinematic parameters of the human hand. Bioengineering (Basel).

[14] Jull G, Amiri M, Bullock-Saxton J (2007). Cervical musculoskeletal impairment in frequent intermittent headache. Part 1: Subjects with single headaches. Cephalalgia.

[15] Yong N, Hu H, Fan X (2012). Prevalence and risk factors for depression and anxiety among outpatient migraineurs in mainland China. J. Headache Pain.

[16] Zang Y, Jiang T, Lu Y (2004). Regional homogeneity approach to fMRI data analysis. Neuroimage.

[17] Charan J, Biswas T (2013). How to calculate sample size for different study designs in medical research?. Indian J. Psychol. Med..

[18] Mingels S, Dankaerts W, van Etten L (2021). Exploring multidimensional characteristics in cervicogenic headache: Relations between pain processing, lifestyle, and psychosocial factors. Brain Behav..

[19] Sedighi A, Nakhostin AN, Naghdi S (2017). Comparison of acute effects of superficial and deep dry needling into trigger points of suboccipital and upper trapezius muscles in patients with cervicogenic headache. J. Bodyw. Mov. Ther..

[20] Huang T, Zhao Z, Yan C (2016). Altered spontaneous activity in patients with persistent somatoform pain disorder revealed by regional homogeneity. PLoS One.

[21] Liu X, Hou Z, Yin Y (2020). CACNA1C gene rs11832738 polymorphism influences depression severity by modulating spontaneous activity in the right middle frontal gyrus in patients with major depressive disorder. Front. Psychiatry.

[22] Medina S, Bakar NA, O'Daly O (2021). Regional cerebral blood flow as predictor of response to occipital nerve block in cluster headache. J. Headache Pain.

[23] Mohamadi M, Rojhani-Shirazi Z, Assadsangabi R (2020). Can the positional release technique affect central sensitization in patients with chronic tension-type headache? A randomized clinical trial. Arch Phys Med Rehabil.

[24] Kim J, Shin J, Oh JH (2015). Longitudinal FDG microPET imaging of neuropathic pain: Does cerebellar activity correlate with neuropathic pain development in a rat model?. Acta Neurochir. (Wien).

[25] Jie W, Pei-Xi C (1992). Discharge response of cerebellar Purkinje cells to stimulation of C-fiber in cat saphenous nerve. Brain Res..

